# Anisotropic chemical expansion due to oxygen vacancies in perovskite films

**DOI:** 10.1038/s41598-021-93968-1

**Published:** 2021-07-27

**Authors:** M. Tyunina, O. Pacherova, T. Kocourek, A. Dejneka

**Affiliations:** 1 Microelectronics Research Unit, Faculty of Information Technology and Electrical Engineering, University of Oulu, P. O. Box 4500, 90014 Oulu, Finland; 2 Institute of Physics of the Czech Academy of Sciences, Na Slovance 2, 18221 Prague, Czech Republic

**Keywords:** Surfaces, interfaces and thin films, Electronic properties and materials

## Abstract

In scientifically intriguing and technologically important multifunctional *AB*O_3_ perovskite oxides, oxygen vacancies are most common defects. They cause lattice expansion and can alter the key functional properties. Here, it is demonstrated that contrary to weak isotropic expansion in bulk samples, oxygen vacancies produce strong anisotropic strain in epitaxial thin films. This anisotropic chemical strain is explained by preferential orientation of elastic dipoles of the vacancies. Elastic interaction of the dipoles with substrate-imposed misfit strain is suggested to define the dipolar orientation. Such elastic behavior of oxygen vacancies is anticipated to be general for perovskite films and have critical impacts on the film synthesis and response functions.

## Introduction

Perovskite-structure *AB*O_3_-type metal oxides are known for their remarkable properties, which include the whole range of electronic states from insulator to superconductor, rich variety of ordering phenomena, and numerous strong responses to external stimuli. High-temperature superconductors, best performing piezoelectrics, large permittivity dielectrics, electro-optic crystals, ionic conductors, and catalysts are just a few examples of multifunctional perovskites. The diverse applications and commercialization of many of these materials have been well established in the fields of electronics and photonics, and the synthesis and development of more advanced oxides for emerging innovative applications are in growing demand. Concurrently, extensive scientific efforts are devoted to fascinating fundamental physics of perovskites.

Comprising 60% of all atoms, oxygen sites are crucial for the phase stability and phenomenal properties of *AB*O_3_ perovskites. Naturally, oxygen vacancies are the most common point defects in these materials. Direct compositional analysis is typically unable to explicitly detect small deviations from oxygen stoichiometry. Instead, the vacancy-induced (or chemical) expansion of the crystal lattice is widely accepted as a sign for the presence of vacancies.

Progress in the synthesis of epitaxial *AB*O_3_ films has opened a new research field related to perovskites^1,2^. A mismatch in lattice parameters, symmetries, and/or thermal expansion coefficients between unstressed film’s material and material of underlying substrate imposes a lattice strain in epitaxial film. This strain leads to unusual phases and functional responses, not available in bulk prototypes^3–5^. Additionally, first-principles calculations showed that strain can reduce the formation energy for oxygen vacancies in epitaxial films, where, in turn, the vacancies can lead to significant changes of electronic properties^6–10^. It is worth noting that the majority of thin-film growth techniques allow for varying oxygen content^1^. Often, however, these techniques also generate unintentional oxygen deficiency. Knowledge on the formation and role of oxygen vacancies is vital in the quest to tailor the functional responses and growth of epitaxial perovskite films.

Here we focus on the poorly studied elastic properties of oxygen vacancies in *AB*O_3_ perovskites. We have previously demonstrated that the elastic interactions of oxygen vacancies with substrate-imposed misfit strain can lead to a special spatial arrangement of the vacancies and thus stabilize epitaxial films of SrTiO_3_ and BaTiO_3_^11–14^. Both SrTiO_3_ and BaTiO_3_ are *A*^2+^*B*^4+^O_3_-type perovskites, where the formation of an oxygen vacancy (V_O_) is associated with strong anisotropic lattice distortions. The nearest to vacancy Ti-cations shift away from the vacancy along the Ti–O-Ti–O-direction. The Ti-Vo-Ti distance elongates compared to the Ti–O-Ti distance^14,15^. This elongation is the primary mechanism, which determines the emergence of stress around the vacancy. The stress is described by an anisotropic elastic dipole tensor (or elastic dipole for brevity), associated with the vacancy^16,17^.

Anisotropic lattice distortions around oxygen vacancies are inherent to many other *AB*O_3_ perovskites^6–10,18,19^. This general property points to the possible universal presence of vacancy-misfit interactions in epitaxial *AB*O_3_-type films. In this work, we examine this possibility by investigating epitaxial films of NdNiO_3_ (NNO). NNO is an *A*^3+^*B*^3+^O3-type perovskite, whose charge transport properties and metal–insulator transition attract much interest. Oxygen vacancies are believed to drive the NNO properties towards the insulating state^9,10^. Similar to the Ti-Vo elongation compared to the Ti–O distance in SrTiO_3_, the Ni-V_O_ distance is longer than the Ni–O distance in NNO^10^. Thus, anisotropic oxygen vacancy dipoles are present in NNO.

To probe vacancy-misfit interactions in NNO, we studied epitaxial NNO films prepared on different substrates enabling either compressive or tensile in-plane strain. Oxygen deficiencies were introduced in the films *in-situ*, by varying the ambient oxygen pressure during pulsed laser deposition. We observed a massive expansion of the unit-cell volume with decreasing pressure, which cannot be rationalized in terms of an isotropic chemical expansion. These observations are ascribed to an anisotropic chemical strain, which arises due to elastic vacancy-misfit interactions. It is further suggested that both the vacancy-misfit interactions, as well as the interactions between vacancy dipoles, can set the specific orientation of the vacancy dipoles in each film. Finally, we discuss the potential impacts of the elastic behavior of the vacancies on the synthesis and response functions of perovskite films.

## Results and discussion

Epitaxial NNO films of ~ 20 nm in thickness were grown by pulsed laser deposition as described before^11–14,20^. To introduce oxygen vacancies, oxygen pressure of deposition, p_O2_, was varied from 20 Pa for stoichiometric growth to as low as 0.1 Pa (see Methods section). We used (001)(La_0.3_Sr_0.7_)(Al_0.65_Ta_0.35_)O_3_ (LSAT) and (001)LaAlO_3_ (LAO) substrates, ensuring cube-on-cube-type coherent growth of NNO and different signs of misfit strain therein. The theoretical biaxial in-plane misfit strain *s*_*a*_ was calculated as *s*_*a*_ = *a*_*SUB*_/*a*_*NNO*_ -1, where *a*_*SUB*_ and *a*_NNO_ = 3.807 Å are the lattice parameters of the substrate and the pseudocubic perovskite cell of NNO, correspondingly. The strain is tensile *s*_*a*_ ≈ 1.6% for NNO on LSAT and compressive *s*_*a*_ ≈ −0.4% for NNO on LAO.

**Figure 1:**
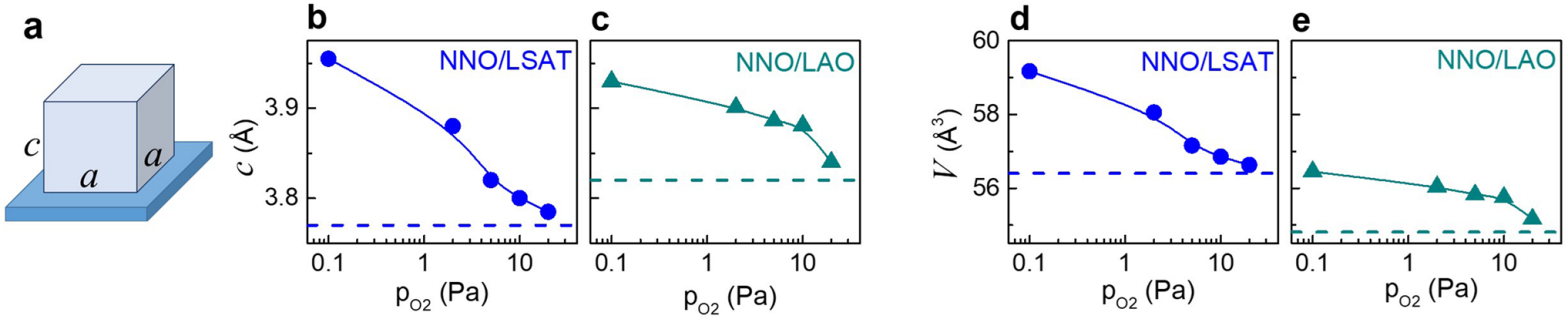
(**a**) Schematics of a pseudocubic perovskite cell of NNO film on top of a cubic substrate. The in-plane lattice parameters *a* and out-of-plane lattice parameter *c* are indicated. (**b,c**) Out-of-plane lattice parameter and (**d,e**) unit cell volume as a function of oxygen pressure for the (**b,d**) NNO/LSAT and (**c,e**) NNO/LAO films. Dashed lines show theoretical values for stoichiometric NNO films.

Structural analyses (see Methods section) revealed good epitaxial NNO/LSAT and NNO/LAO coherence for all pressures except 0.1 Pa (Supplementary Figs. S1–S3). Pseudocubic perovskite cells of NNO are aligned in a cube-on-cube manner with underlying substrates, i.e., the films’ (00* l*) planes are parallel to the (001) substrate planes (substrate surface) and the films’ [100] (and [101]) crystal directions are parallel to those in the substrates (Fig. 1a).

The out-of-plane lattice parameter *c* is found to significantly increase with decreasing oxygen pressure for both the NNO/LSAT and NNO/LAO films (Fig. 1b, c). The unit cell volume *V* increases as well (Fig. 1d, e). For the 20 Pa pressure, the parameter *c* and volume *V* are close to those theoretically estimated in stoichiometric NNO (Supplementary section S2). Compared to unstressed stoichiometric NNO, the relative volume expansion is massive at low pressures (up to 5%).

Although unintentional formation of inclusions of Ruddlesden–Popper phase and associated local elongation of the out-of-plane lattice parameter may occur, such inclusions do not produce an overall out-of-plane elongation in epitaxial films of nickelates^21–23^. Concurrently, a hypothetical elongation due to dominating fraction of the Ruddlesden–Popper phase should have been accompanied by the corresponding additional x-ray diffraction peaks^24^, which is not the case here (Supplementary section S1).

**Figure 2:**
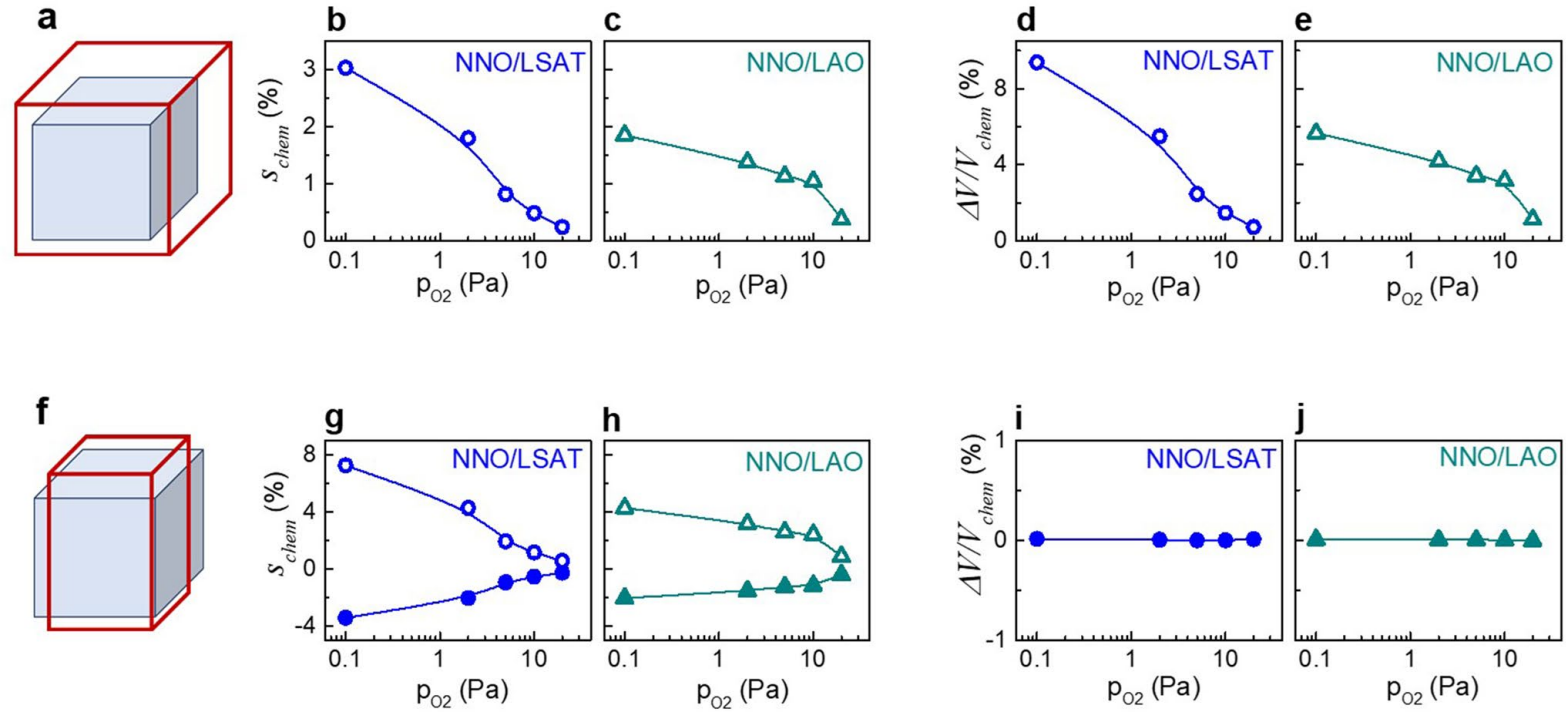
Analysis of (**a–e**) isotropic and (**f–j**) anisotropic chemical expansion in epitaxial NNO films. (**a,f**) Schematics of perovskite unit cells in oxygen-deficient unstressed material of the films (red lines) compared to stoichiometric bulk prototype (blue cubes). (**b,c,g,h**) Estimated chemical strain and (**d,e,i,j**) volumetric chemical expansion as a function of oxygen pressure. In (**g,h**), open symbols show the out-of-plane strain, whereas solid symbols show the in-plane strain, correspondingly.

We note that the observed pressure-dependent lattice expansion, which is manifested in the increase of the parameter *c* and unit-cell volume *V*, cannot be directly interpreted as a chemical expansion (or chemical strain) because both the chemical strain and misfit strain are present in the films. To extract the chemical strain from the measured parameters, we assumed an oxygen-deficient material, which is then subjected to a substrate-controlled stress for each film. We considered two cases: a conventional isotropic chemical expansion, at which the material’s cell is always metrically cubic and all lattice parameters change similarly with pressure (Fig. 2a), and an unconventional specific anisotropic chemical expansion, at which the material’s metrical tetragonality is a function of pressure (Fig. 2f). Using the measured lattice parameters and epitaxial elastic relationships (Supplementary sections S2 and S3), we extracted the lattice parameters, chemical strain, and volumetric chemical expansion of the unstressed material for the isotropic and anisotropic cases (Fig. 2).

In the isotropic case, we obtained a gigantic chemical strain *s*_*chem*_ of ~ 2% (Fig. 2b, c). This implies a nonsensical oxygen deficiency *δ* > 10 for typical coefficients of isotropic chemical expansion in *AB*O_3-δ_^16,17^. Additionally, such a huge strain cannot be produced by the hypothetical presence of cationic vacancies.

In the anisotropic case with the volumetric chemical expansion set to zero (Fig. 2i, j), we extracted comparable chemical strains for the unstressed materials from the NNO/LSAT and NNO/LAO data (Fig. 2g, h). The chemical strain is tensile in the out-of-plane direction and compressive in-plane. By setting the volumetric chemical expansion to 1%, somewhat smaller magnitudes of the chemical strain can be obtained, but the presence of anisotropy is stable (Supplementary Fig. S5). Importantly, in contrast to the huge volumetric expansion in the isotropic case, only minor volumetric expansion, if any, occurs in the anisotropic case.

We emphasize that isotropic chemical strain cannot rationally explain these experimental observations. In contrast, an anisotropic chemical strain is likely, which can be related to the vacancy-misfit interactions as follows. In *AB*O_3_ perovskite, the elastic dipole of oxygen vacancy V_O_ is governed by the lowered symmetry due to the *B*-V_O_-*B* elongation (see Supplementary section S4 and Fig. S6 therein). This simplified dipole is a second rank tensor with non-zero diagonal components: *D*_*1*_ along the *B*-V_O_-*B* direction and *D*_*2*_ = *D*_*3*_ orthogonal to the *B*-V_O_-*B* direction. For instance, in the typical cubic perovskite SrTiO_3_, the components are *D*_*1*_ > 0, *D*_*2*_ = *D*_*3*_ < 0, and *D*_*1*_ >|*D*_*2*_ |^16^. In contrast to oxygen vacancies, dipoles of cationic vacancies (V_A_ or V_B_) are isotropic. Although anisotropic dipoles may also arise due to oxygen-cation divacancies (V_A_-V_O_ or V_B_-V_O_), their occurrence is obviously less probable (compared to V_O_) and omitted for simplicity.

**Figure 3:**
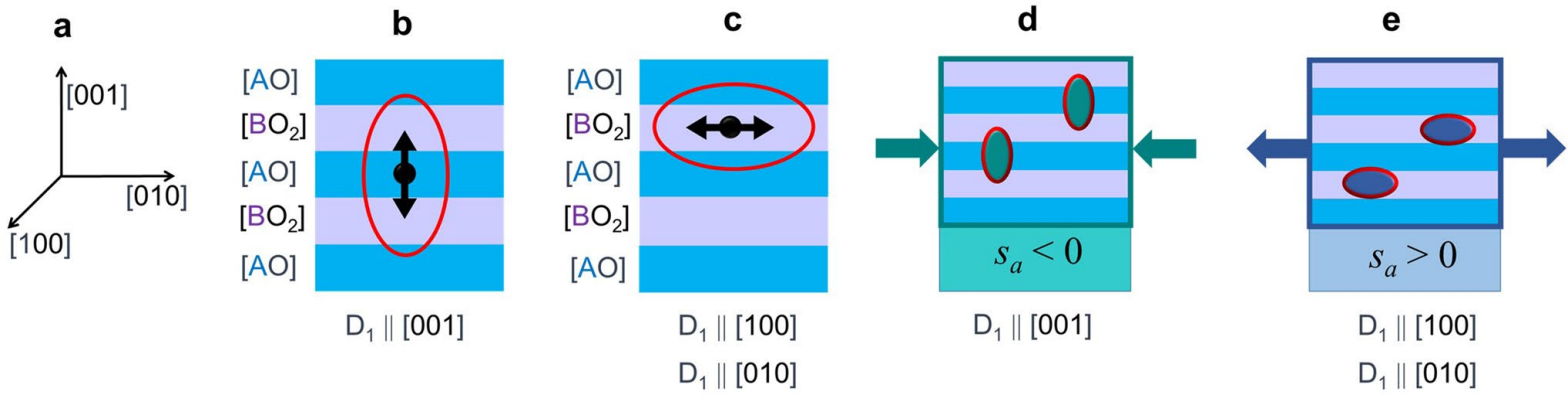
(**a–c**) Schematics of elastic dipoles of oxygen vacancies for (**b,c**) vacancies in different atomic planes with respect to (**a**) crystal directions in *AB*O_3_ perovskite. (**d,e**) Schematics of elastic dipoles of oxygen vacancies in strained cube-on-cube epitaxial films.

Importantly, an *AB*O_3_ crystal can be thought of as a periodical arrangement of the (001)[*A*O] and (001)[*B*O_2_] atomic planes (Fig. 3a–c). Correspondingly, a vacancy formed in the (001)[*A*O] plane will have a dipole oriented along the [001] crystal direction: *D*_*1*_ || [001] (Fig. 3b). For the vacancy in the (001)[*B*O_2_] plane, two dipole orientations are possible: *D*_*1*_ || [100] and *D*_*1*_ || [010] (Fig. 3c). It is worth mentioning that in perovskites with lower (than cubic) crystal symmetry, the magnitudes of the *B*-Vo-*B* elongations and, hence, of the dipole components can differ for vacancies in different atomic planes^9,15^. However, this difference does not affect the dipolar orientation, which here is of central importance.

**Figure 4:**
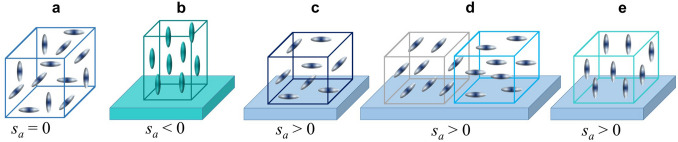
Schematics of orientations of elastic dipoles of oxygen vacancies in (**a**) bulk unstressed sample, (**b**) compressively strained epitaxial film, and (**c–e**) tensile strained epitaxial film.

Oxygen vacancies are randomly distributed in bulk unstressed crystals, where consequently, each vacancy dipole is randomly oriented (Fig. 4a). When averaged over the entire crystal, however, the relative elongation of the bulk lattice parameters caused by all vacancies (or chemical strain) is isotropic. The typical coefficients of such isotropic chemical expansion are small, where for a very strong oxygen deficiency *δ* = 1 in *AB*O_3-δ_, the expected chemical strain is only about 0.1–0.2%^16,17^.

In cube-on-cube-type (001)-oriented epitaxial films, the [*A*O] and [*B*O_2_] atomic planes are parallel to the substrate (001) surface. Hence, if a vacancy is located in the (001)[*A*O] plane, its dipole will be out-of-plane, oriented normal to the substrate surface, whereas vacancies in the (001)[*B*O_2_] plane will have dipoles oriented in-plane (Fig. 3a–c). In contrast to unstressed bulk crystals, epitaxial films experience a biaxial in-plane stress imposed by their substrates (as shown schematically with arrows in Fig. 3d, e). The dipoles interact with the substrate-induced strain. The dipole-strain interaction energy depends on the dipole’s orientation (Supplementary section S4). For an out-of-plane dipole, the interaction energy *E*_*DC*_ is.

1 $$ E_{DC} = - s_{a} \left( { - \frac{{2c_{12} }}{{c_{11} }}D_{1} + 2D_{2} } \right), $$

where *c*_12_ and *c*_11_ are the elastic constants of the film material. For an in-plane dipole, the interaction energy *E*_*DA*_ is.

2 $$ E_{DA} = - s_{a} \left( {D_{1} + D_{2} \left( {1 - \frac{{2c_{12} }}{{c_{11} }}} \right)} \right). $$

As seen from (1–2), the energies are *E*_*DC*_ < *E*_*DA*_ for *s*_*a*_ < 0 and *E*_*DA*_ < *E*_*DC*_ for *s*_*a*_ > 0. In other words, the out-of-plane dipole orientation is promoted by a compressive in-plane strain (Fig. 3d), whereas the in-plane orientation is promoted by a tensile strain (Fig. 3e). Interestingly, the energies for vacancy formation in different atomic planes may differ in magnitude by ~ 10%^8,10,15^, but the energies *E*_*DC*_ and *E*_*DA*_ can differ dramatically because of their signs^14^. Thus, the elastic properties, rather than the formation energy, can determine a type of the atomic planes, where vacancies are located in epitaxial films.

Contrary to random orientations of vacancy dipoles in an unstressed bulk sample (Fig. 4a), the out-of-plane dipoles are preferable in compressively strained films (Fig. 4b). As a consequence of such preferential dipolar orientation, the film elongates in the out-of-plane direction and an anisotropic chemical strain appears. The detected behavior of the NNO films on compressive LAO substrates (Fig. 2h) agrees perfectly with this scenario. Notably, the NNO/LAO behavior is consistent with the out-of-plane dipolar orientation in compressively strained films of SrTiO_3_ and BaTiO_3_, where in addition to the lattice expansion, an internal electric field also evidenced the dipolar orientation^11–13^. The out-of-plane dipoles correspond to vacancies in the [NdO] planes in NNO (Fig. 3b, d). The out-of-plane chemical strain of ~ 4% may result from an oxygen deficiency of *δ* ≈ 0.5 (Supplementary section S5).

The out-of-plane dipolar orientation induced by compressive misfit is unambiguous and similar for all dipoles (Fig. 4b), which also minimizes dipole–dipole interactions. However, a tensile strain may lead to two different orthogonal in-plane dipolar orientations. Whereas random distribution of differently oriented in-plane dipoles enhances dipole–dipole interactions (Fig. 4c), arrangements of the in-plane dipoles in ordered regions may weaken these interactions but concurrently yield boundaries (Fig. 4d). Although effects of the dipole–dipole interactions are unstudied, it is likely that in-plane dipoles (Fig. 4c, d) are less stable than out-of-plane dipoles (Fig. 4e). Here, the out-of-plane dipolar orientation is found in the tensile strained NNO/LSAT films. We emphasize that the bulk-like random dipolar orientation (Fig. 4a) cannot explain lattice expansion in these films. We also emphasize the difference between the bulk (*s*_*a*_ = 0) and thin-film (*s*_*a*_ < 0) elastic systems (compare Fig. 4a and e).

A thorough multiscale theoretical analysis is needed to clarify the exact mechanisms for preferential orientations of oxygen vacancy dipoles in strained films. Our experimental observations indicate such preferential orientation. We note that the vacancy dipoles and elastic constants are not exactly known for NNO. However, our conclusions remain valid if these parameters are simply within the same order of magnitude as used in the estimations. This condition is most likely satisfied for many *AB*O_3_ perovskites. We believe that anisotropic chemical strain due to oxygen vacancy dipoles is general for epitaxial films of these materials.

Importantly, anisotropic chemical strain is only one of many possible effects produced by elastic interactions of vacancy dipoles with an epitaxial misfit strain. For instance, the out-of-plane dipoles can reduce elastic energy in compressively strained films and thus lead to an enlarged critical thickness for the relaxation of the misfit strain. Hence, relatively thick strained epitaxial films can be stabilized^14^. Moreover, a very large combined misfit-chemical strain can be achieved. Elastically defined preferential locations of oxygen vacancies in a definite type of the atomic planes can lead to vacancy ordering, new phases, or phase coexistence /separation. Because elastically defined vacancy locations are invariable in strained epitaxial films, the migration of oxygen vacancies is therein prohibited: a restriction that is critical for charge transport properties^25^. Due to strain-polarization coupling in piezoelectric and ferroelectric perovskites, there are electric dipoles associated with elastic dipoles. Thus, strain-controlled preferential dipolar orientation can create an internal electric field, that alters functional responses^11,12^. A far-reaching number of effects may exist beyond these given examples. To predict, employ, or avoid these effects, the atomic-level nature and appropriate macroscopic-level parameters of oxygen vacancies, as well as the mechanisms of their elastic behavior in epitaxial films should be better understood. Multiscale theoretical modeling is of preeminent importance in this field.

## Conclusions

It is demonstrated that oxygen vacancies produce anisotropic chemical strain in epitaxial films of perovskite oxide NdNiO_3_. This is in contrast to isotropic chemical strain in bulk unstressed perovskites. It is suggested that elastic interactions of oxygen vacancy dipoles with substrate-imposed misfit strain can stabilize the preferential orientation of the vacancy dipoles and lead to an anisotropic chemical strain in cube-on-cube-type epitaxial perovskite films. This elastic behavior of oxygen vacancies is anticipated to induce many other essential effects in epitaxial films and deserves further investigations.

## Methods

Thin films of NNO were deposited using single-crystal epitaxially polished (001)LSAT and (001) LAO substrates purchased from MTI Corporation. The films were grown by pulsed laser deposition using a KrF excimer laser (energy density ~2 J/cm^2^). A substrate temperature of 973 K was kept during deposition and lowered at a rate of 5 K·min^−1^ during post-deposition cooling. The oxygen pressure was kept constant during deposition and post-deposition cooling, and varied from 0.1 to 20 Pa for different samples. The NNO/LSAT and NNO/LAO films were prepared in pairs, within the same deposition process. The thickness of the films was regulated by number of laser pulses. For electrical characterization, Au electrodes were prepared by pulsed laser deposition as well.

The crystal structure of the films was studied by high-resolution x-ray diffraction on a D8 DISCOVER diffractometer (Bruker corporation) using Cu Kα radiation. The lattice parameters were estimated from the positions of the diffraction maxima using the substrates as a reference. The thickness of the films was determined from the positions of the Laue satellites. The diffraction data were fitted using LEPTOS software.

## Supplementary Information

Supplementary Information.

## Data Availability

The datasets generated and/or analyzed during the current study are available from the corresponding author on reasonable request.
