# Supplementary material for: Anisotropic chemical expansion due to oxygen vacancies in perovskite films
Source: Sci Rep. 2021 Jul 27;11:15247. doi: 10.1038/s41598-021-93968-1 (PMC8316387; doi:10.1038/s41598-021-93968-1)
Supplement: Supplementary file 1 — Supplementary Information. [file 41598_2021_93968_MOESM1_ESM.pdf]

Supplementary information

to

Anisotropic chemical expansion due to oxygen vacancies in perovskite films

M. Tyunina,[1,2,*] O. Pacherova,[2] T. Kocourek,[2] A. Dejneka[2]

[1]Microelectronics Research Unit, Faculty of Information Technology and Electrical Engineering, University of Oulu, P. O. Box 4500, FI-90014 Oulu, Finland

[2]Institute of Physics of the Czech Academy of Sciences, Na Slovance 2, 18221 Prague, Czech Republic

*E-mail: marina.tjunina@oulu.fi

S1. Crystal structure.

S2. Epitaxy and elastic relationships.

S3. Chemical expansion.

S4. Elastic dipole tensor of oxygen vacancy.

S5. Oxygen deficiency.

S1. Crystal structure.

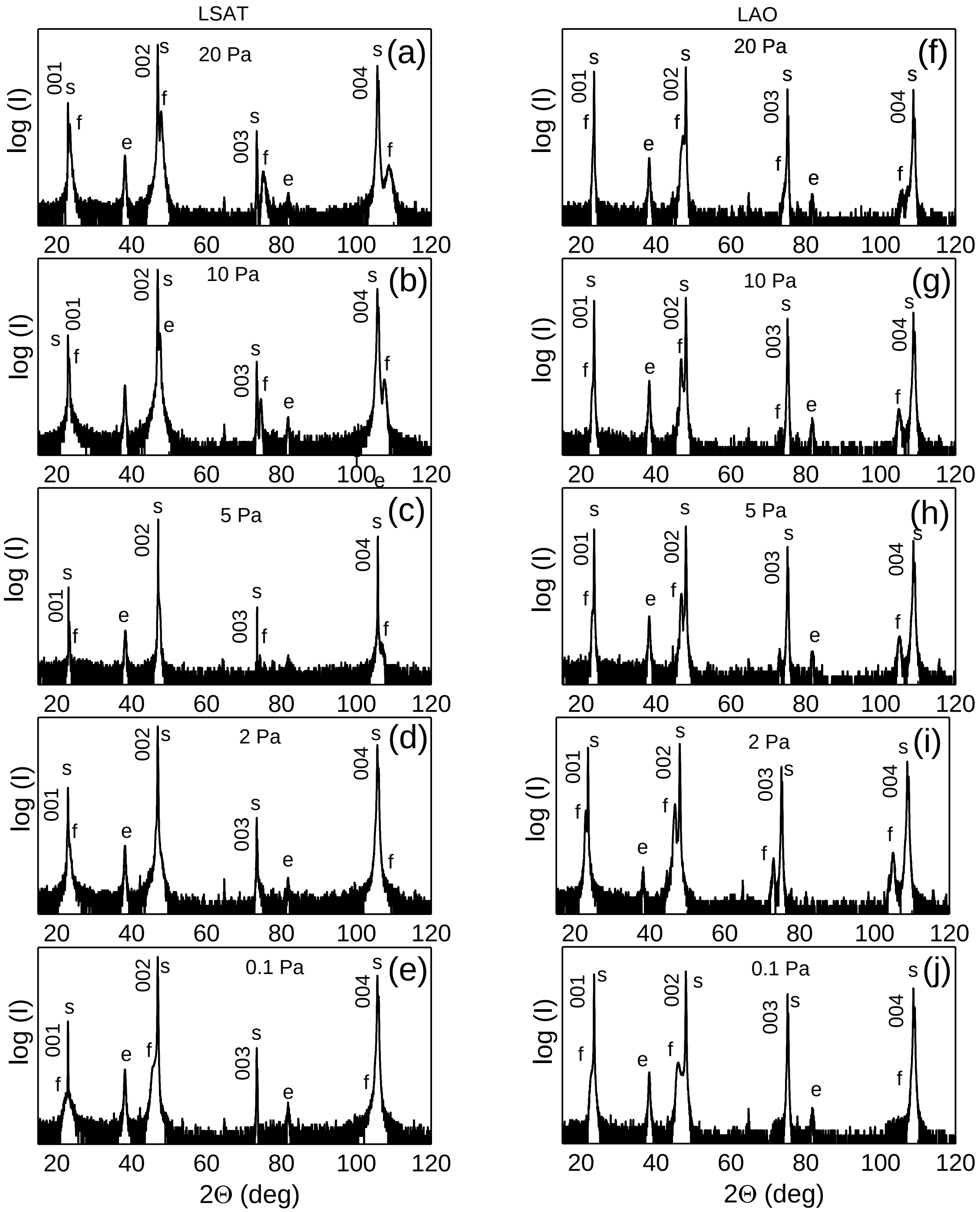

Fig. S1. XRD Θ-2Θ scans in the (a-e) NNO/LSAT and (f-j) NNO/LAO films deposited at different pressures as marked on the plots. Diffraction peaks from the substrates, films, and Au electrodes are denoted by s, f, and e, correspondingly.

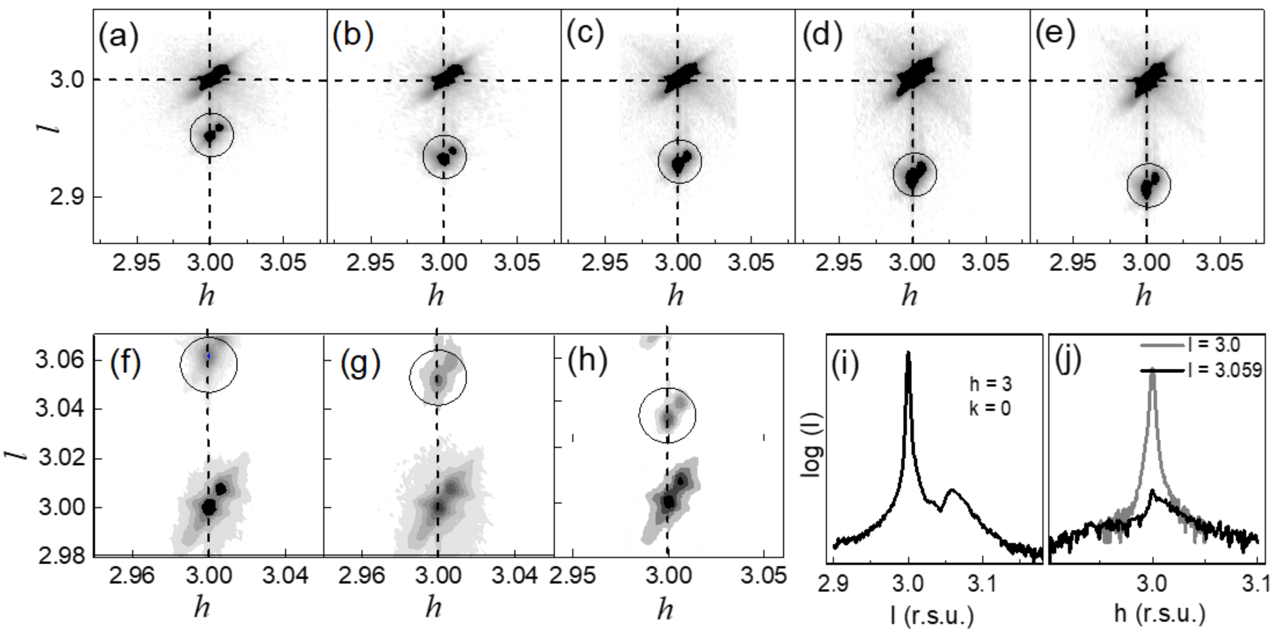

Fig. S2. Typical reciprocal space (a-h) maps and (i, j) scans around (303) reciprocal lattice point in the (a-e) NNO/LAO and (f-j) NNO/LSAT. The indices are expressed in reciprocal lattice units of the substrates. Intensity scale is selected to better illustrate film-substrate coherency. Circles mark diffractions from the films and dashed lines show *h*-alignment between the films and substrates in (a-h). Deposition pressure was (a, f) 20, (b, g) 10, (c, h) 5, (d, i, j) 2, and (e) 0.1 Pa.

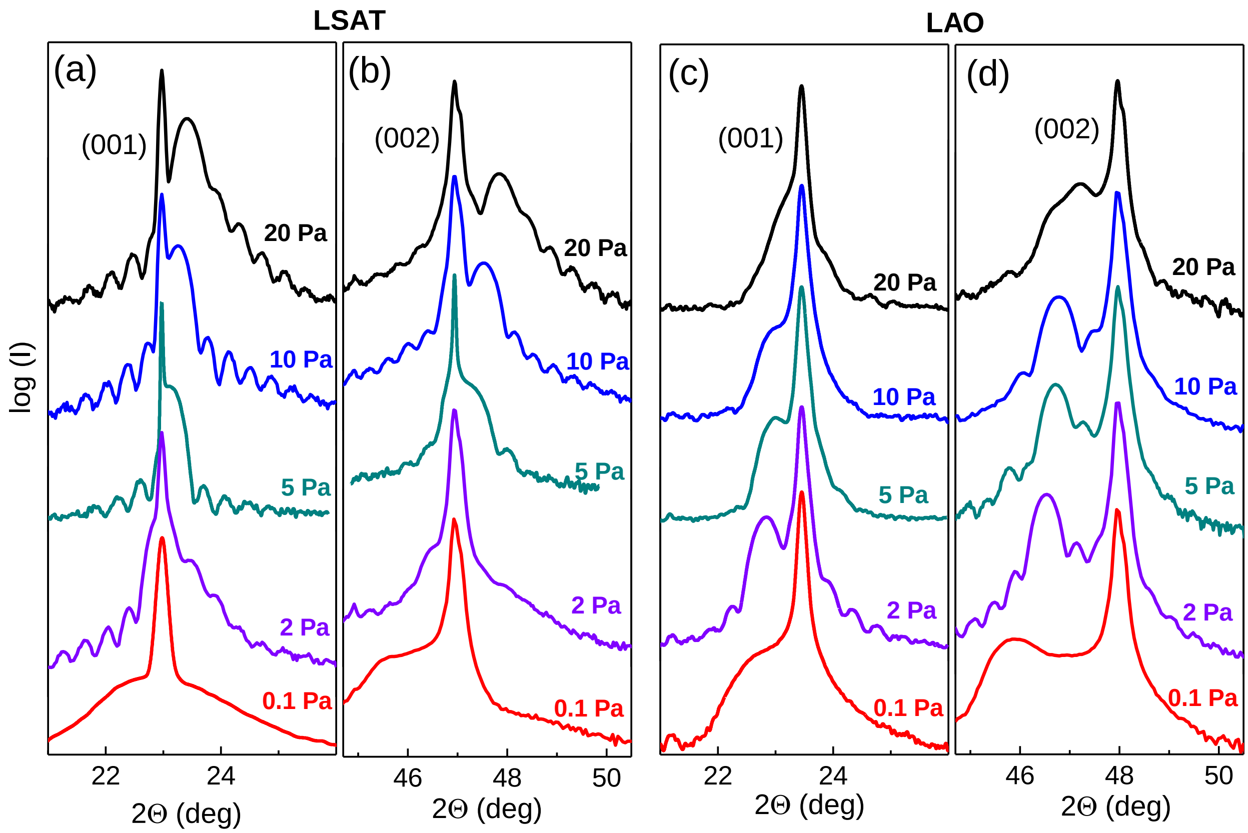

Fig. S3. Details of XRD Θ-2Θ scans around (a, c) (001) and (b, d) (002) peaks in the (a-b) NNO/LSAT and (c-d) NNO/LAO films deposited at different pressures as marked on the plots.

S2. Epitaxy and elastic relationships.

During epitaxial growth, a thin film of a material is formed on the top of a substrate, whose crystal symmetry and lattice parameters may differ from those of the material. Here, first, a cube-on-cube epitaxy of a cubic material (lattice parameter a_M) on a cubic substrate (lattice parameter a_{SUB}) is considered [Fig. S4(a)]. Because of the material-substrate mismatch in lattice parameters, the substrate imposes biaxial in-plane compressive stress on the material, whereas there is no out-of-plane stress [Fig. S4(b)]. For a coherent film, where the in-plane lattice parameters a equals to those of the underlying substrate, the substrate-imposed stress leads to a new out-of-plane lattice parameter c, which differs from a_M in the material [Fig. S4(c)]. The lattice parameters, tetragonality, and unit-cell volume in the mechanically stressed (strained) film differ from those in the material and can be estimated as follows.

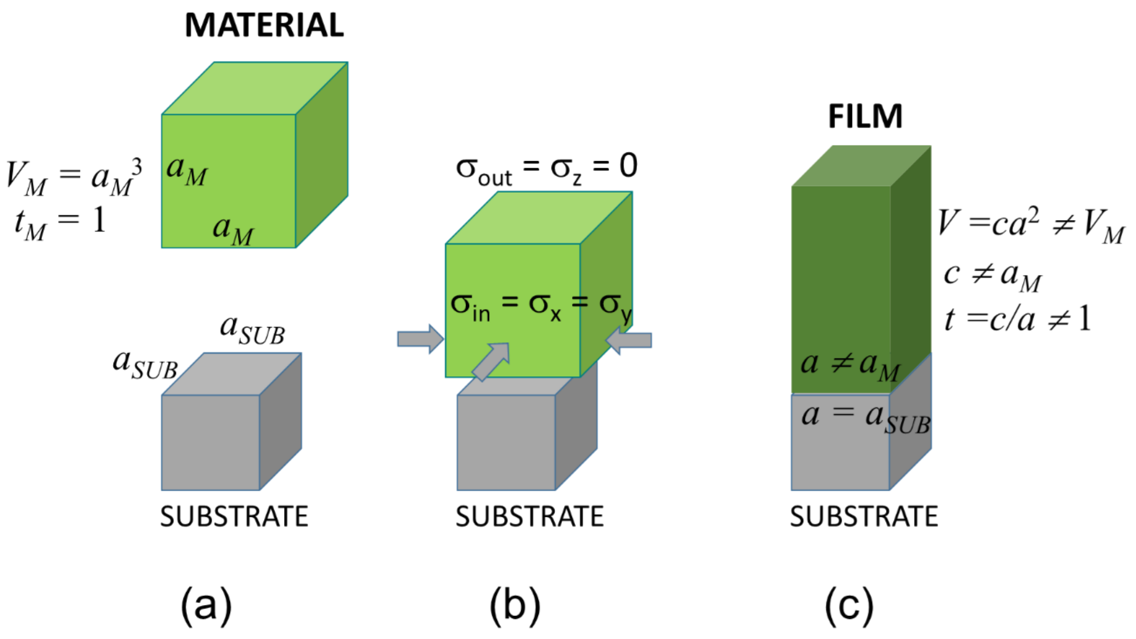

Fig. S4. Schematics of cube-on-cube epitaxy. (a) Unit cells of the material and substrate. (b) Substrate-imposed mechanical conditions. (c) Unit cell of the strained film.

The theoretical material-substrate misfit strain is

$$s_a = \frac{a_{SUB}}{a_M} - 1. \tag{S1}$$

For the coherent strained cube-on-cube film, the in-plane lattice parameters are similar:

$$a = b = a_{SUB} \qquad \text{(S2)}$$

The out-of-plane strain (s_c) and the out-of-plane lattice parameter (c) are elastically related to the in-plane strain (s_a):

$$s_c = -\frac{2c_{12}}{c_{11}} s_a \qquad \text{(S3)}$$

$$c = a_M(1 + s_c) = a_M\left(1 - \frac{2c_{12}}{c_{11}} s_a\right) \quad . \qquad \text{(S4)}$$

Here c_{11} and c_{12} are the elastic constants of the material. For NNO, we used data from [1].

The unit-cell volume V and tetragonality t of the strained film are, correspondingly:

$$V = ca^2 = a_0^3(1 + s_a)^2\left(1 - \frac{2c_{12}}{c_{11}} s_a\right) \quad , \qquad \text{(S5)}$$

$$t = \frac{c}{a} = \frac{\left(1 - \frac{2c_{12}}{c_{11}} s_a\right)}{(1 + s_a)} \quad . \qquad \text{(S6)}$$

The relationships (S3-S6) make it possible to calculate theoretical lattice parameters of the strained film. They also allow for determining the lattice parameter a_M of an unknown cubic material using the lattice parameters a and c, which are experimentally measured in the film made of such material.

Next, we consider that the unit cell of a material, from which the film is made, can be tetragonal with the lattice parameters (a_M, c_M), tetragonality (t_M), and unit-cell volume (V_M):

$$c_M = t_M a_M \qquad \text{(S7)}$$

$$V_M = t_M a_M^3 \qquad \text{(S8)}$$

The unit-cell volume in a coherent cube-on-cube-type film is V :

$$V = ca^2 = t_M a_M^3 (1 + s_a)^2\left(1 - \frac{2c_{12}}{c_{11}} s_a\right) \qquad \text{(S9)}$$

$$\frac{V}{V_M} = (1 + s_a)^2\left(1 - \frac{2c_{12}}{c_{11}} s_a\right) \qquad \text{(S10)}$$

Or in another form:

$$\left(\frac{V}{V_M} - 1\right) = \left(2 - \frac{2c_{12}}{c_{11}}\right) s_a + \left(1 - \frac{4c_{12}}{c_{11}}\right) s_a^2 - \frac{2c_{12}}{c_{11}} s_a^3 \qquad \text{(S11)}$$

For an unknown material, the strain s_a can be found using expression (S11) and assuming no changes of the material's unit cell volume compared to its cubic prototype. Then the tetragonality of the material is:

$$t_M = \frac{c(1 + s_a)}{a\left(1 - \frac{2c_{12}}{c_{11}} s_a\right)} \quad . \qquad \text{(S12)}$$

Consequently, the lattice parameters of the material can be found from the measured lattice parameters of the film.

S3. Chemical expansion.

Using the lattice parameters extracted for the unstressed oxygen deficient material, the chemical strain is calculated

$$s_{chem} = \frac{a_M}{a_{NNO}} - 1, \ \ s_{chem} = \frac{c_M}{a_{NNO}} - 1, \qquad \text{(S13)}$$

where the lattice parameter of pseudocubic stoichiometric NNO is a_{NNO} = 3.807 Å.

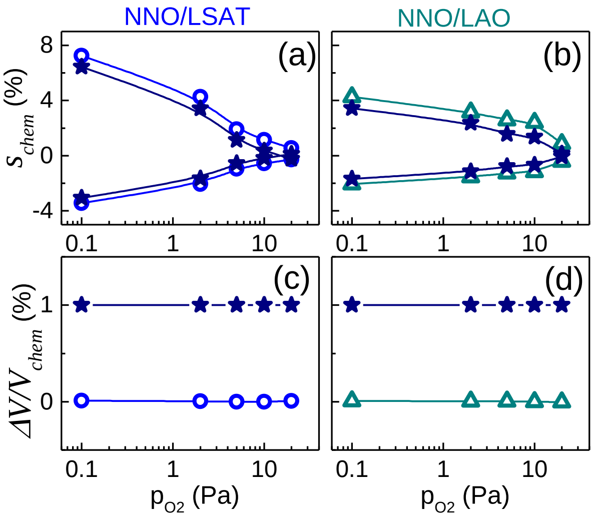

Fig. S5. (a-b) Estimated chemical strain and (c-d) volumetric chemical expansion as a function of oxygen pressure in the anisotropic case. Open symbols correspond to $\Delta V/V = 0$ and solid stars – to $\Delta V/V = 1\%$. The upper (lower) curves show the out-of-plane (in-plane) strain in (a, b).

S4. Elastic dipole tensor of oxygen vacancy.

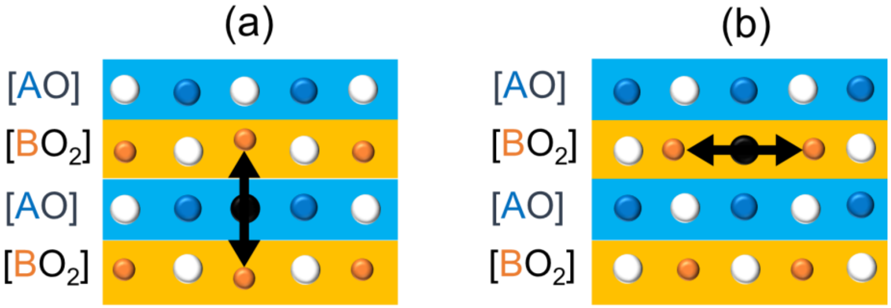

Fig. S6. Schematics of atomic positions in ABO_3 perovskite oxide: A is shown by blue circles, B – by orange circles, and oxygen – by white circles. Atomic planes are highlighted by different colors. Oxygen vacancies are shown by black circles. The vacancy-associated elongation of the B-B distances is shown by black arrows. In (a), vacancy is located in the [AO] plane, and in (b) – in the [BO_2] plane.

Oxygen vacancy possesses anisotropic dipole tensor p_{ij} with non-zero diagonal terms:

$$p_{ij} = \begin{bmatrix} D_1 & 0 & 0 \\ 0 & D_2 & 0 \\ 0 & 0 & D_3 \end{bmatrix}. \qquad \text{(S14)}$$

Here the large positive component D_1 corresponds to elongation along the B-V_O-B direction and smaller similar negative components D_2 and D_3 – to shrinkage in the directions orthogonal to the B-V_O-B direction. Vacancy elastic dipoles interact with a strain field s_{ij}. The dipole-strain interaction energy is [2]

$$E_{ps} = -p_{ij}s_{ij} \,. \qquad \text{(S15)}$$

In a cube-on-cube (001) oriented epitaxial film, strain includes biaxial in-plane strain s_a and out-of-plane strain s_c. Then the dipole-strain interaction energy can be specified for different locations of oxygen vacancies as follows.

For a vacancy in the (001)[AO] plane, which is parallel to the substrate surface, the dipole is oriented so that the component D_1 is in the out-of-plane direction. The interaction energy E_{DC} is

$$E_{DC} = -\begin{bmatrix} D_1 & 0 & 0 \\ 0 & D_2 & 0 \\ 0 & 0 & D_3 \end{bmatrix} \times \begin{bmatrix} s_c \\ s_a \\ s_a \end{bmatrix} = -(D_1 s_c + 2D_2 s_a) = -2s_a\left(D_2 - \frac{c_{12}}{c_{11}} D_1\right). \qquad \text{(S16)}$$

For a vacancy in the (001)[BO_2] plane, which is parallel to the substrate surface, the dipole is oriented so that the component D_1 is in either of the in-plane directions. The dipole-strain interaction energy E_{DA} is

$$E_{DA} = -\begin{bmatrix} D_3 & 0 & 0 \\ 0 & D_2 & 0 \\ 0 & 0 & D_1 \end{bmatrix} \times \begin{bmatrix} s_c \\ s_a \\ s_a \end{bmatrix} = -(D_3 s_c + D_2 s_a + D_1 s_a) = -s_a \left(D_1 + D_2 \left(1 - \frac{2c_{12}}{c_{11}} \right) \right). \quad \text{(S17)}$$

S5. Oxygen deficiency.

The out-of-plane chemical strain, extracted from the experimental data, is rather large, to 4–8 %. To check feasibility of such strain magnitude, we considered that the out-of-plane chemical strain is induced by the out-of-plane dipoles with the concentration N_{DC} and roughly equals to [$s_{chem} \approx (D_1 N_{DC})/c_{11}$]. For $D_1 \approx 5$ eV [3], the estimated $N_{DC} \approx 0.9\text{-}1.8\times10^{28}$ m^{-3} is reasonable compared to the concentration of oxygen atoms ~5.4×10^{28} m^{-3} in stoichiometric NNO.

References

1. Š. Masys, V. Jonauskas, Elastic properties of rhombohedral, cubic, and monoclinic phases of $LaNiO_3$ by first principles calculations, Comput. Mater. Sci. **108,** 153 (2015).
2. E. Clouet, C Varvenne, T. Jourdan, Elastic modeling of point-defects and their interaction, Comput. Mater. Sci. **147,** 49 (2018).
3. D. A. Freedman, D. Roundy, T. A. Arias, Elastic effects of vacancies in strontium titanate: Short- and long-range strain fields, elastic dipole tensors, and chemical strain, Phys. Rev. B **80**, 064108 (2009).
